# Incidence of retromolar canal in mandible using CBCT: An institution based retrospective study

**DOI:** 10.6026/973206300213506

**Published:** 2025-10-31

**Authors:** Thangam K., Anitha R., Sarswathi Gopal K.

**Affiliations:** 1 Department of Oral Medicine and Radiology, Meenakshi Ammal Dental College, Chennai, India

**Keywords:** Cone beam computer tomography, retro molar canal, retro molar foramen, incidence, anatomical variance

## Abstract

Retromolar canal (RMC) is an anatomic structure located in the Retro molar area distal to the third mandibular molar. The retro molar area is considered as an imperative site where many dental surgeries are being held and due to increasing demand for surgical procedure in the retromolar area of the mandible. The identification of the retromolar canal has become an issue of clinical concern. Therefore, it is of interest to determine the incidence of RMC in the mandible using CBCT and measure the distance between the Retro molar Foramen. Hence, 101 CBCT scans were examined to detect the presence of RMC on 3D CBCT. Data shows that the RMC was present in 64 out of 101 scans with 63.3% incidence rate.

## Background:

The mandible, which is the most significant and strongest bone in the face, is an intricate bone in terms of both its morphology and development. It contains nerve and vascular supply and serves as an anchor for the lower teeth [1]. Comprehending the human mandible's shape and potential changes is crucial for organizing various dental operations, such as orthognathic surgery, mandibular reconstruction, third molar extraction and dental implant insertion [2, 3]. RMF is referred to as the mandibular Retro molar canal (RMC) apertures and it is clinically significant because a neurovascular bundle passes through it [4, 5]. The Retro molar trigone, which is bounded anteriorly by the third molar, medially by the temporal crest and laterally by the anterior border of the ramus, contains the RMF, which is situated posteriorly to the last molar [6]. According to cadaveric dissections, the Retro molar canal (RMC) curves in a postero -superior direction behind the third molar to open into the Retro molar foramen. It branches off the main mandibular canal and follows a recurring route [7]. It comprises of Neurovascular bundles, which consist of several arteries, venules and myelinated nerve fibers [8, 9, 10, 11- 12]. However the components of an RMC haven't been precisely specified, though. The canal may contain aberrant buccal nerves or convey additional innervations to the mandibular teeth [10, 11]. In 1967, Schejtman *et al.* was the first to describe the existence of a Retro molar canal; Ossenberg followed suit in 1987 [13, 14]. The incidence of Retro molar canals and foramina varies greatly, according to various authors, with numbers ranging from 12% to 75% for canals and 0 to 52% for foramina [13, 15]. Therefore, it is of interest to determine the incidence and prevalence of RMC in south Indian population.

## Materials and Methods:

This study employed 101 CBCT volumes between the age of 16 to 60yrs of age was acquired from the dental archives that were generated using Planmeca Promax 3D mid proface CBCT machine and assessed with Romexis software. Full skull Images with good contrast and large field-of view (FOV) scan showing the entire retro molar region of the mandible bilaterally are included in this study. Images with artefacts, radiologic evidence of any bony injury, any surgical intervention in the retromolar region of the mandible, presence of any obvious jaw bone anomaly/pathology in the retromolar region, the presence of an impacted third molar were excluded in this study. Multi-planner reconstruction (MPR) was scrolled to identify the presence of RMF or RMC. Scans were examined thoroughly for the presence of any opening on the outer surface of the mandible in the retro molar area, which may indicate the RMF or any branching from the inferior alveolar canal. Cross sections, reconstructed panoramic view and reconstructed slice panoramic view were also scrolled thoroughly in arch-section module to facilitate tracing of the inferior alveolar canal (IAC) and identify any sign of branching in the retro molar area from the IAC indicating the presence of RMC. When the RMF or RMC was identified, linear measurements were done and recorded for statistical analysis. Sagittal cut was adjusted to be passing through the RMF and the middle of the 3rd molars, and then, linear measurement was done in the anteroposterior direction. Axial cut was adjusted to be passing through the RMF and the mandibular foramen and the linear measurements were done between them in both anteroposterior direction.

All RMCs were classified according to their course and direction to three main types according to Patel *et al.* classification:

[1] Type A in which the RMC branches from the mandibular canal distal to the third molar and course superiorly to open in the retromolar area with RMF. Two variations from type A were noted A1 and A2. In A1, the canal runs in a straight manner superiorly to open in the retromolar area; however, in A2, the canal runs in a curved manner anteriorly then postero-superiorly to open the retromolar area.

[2] Type B in which the canal starts from the retromolar area by the RMF and ends in the radicular portion of the 3rd molar.

[3] Type C in which the canal starts branching early from the mandibular canal near the mandibular foramen and ends in a higher level in the retromolar area.

## Results:

The data obtained was entered in Microsoft Excel Spreadsheet and was subjected to statistical analysis. All analyses were carried out using Statistical Package for Social Sciences (version 26, IBM, Chicago, USA). Descriptive statistics was done using Frequency (n and %) distribution and inferential statistics was done using a chi-squared test. A p-value of <0.5 was considered to be statistically significant. The sample consisted of 101patient scans, 65 males and 35 females. The results showed the presence of 42 RMCs in males and 22 RMCs in females with a total of 64 patients having RMCs out of 101 and an incidence rate of 63.3% (Table 1 - see PDF). According to the 202 right and left sides in the total sample size, the results showed the presence of 64 RMCs in males and 30 RMCs in females with a total of 94 RMCs out of 428 sides with an incidence rate of 46.5% (Table 2 - see PDF). The incidence of the different types of RMCs was evaluated, Type C is found to be the most common type (n =53, 56.3%) and type A was found to be 2nd most common type (n = 20, 21%) which has two subtypes A1 and A2. Type B was the rarest type (n = 8, 8%) (Table 3 - see PDF). The prevalence of RMCs on the left side (n = 41, 20.2%) was slightly lower than that of the right side (n = 53, 26.2%), with a significant difference (Table 4 - see PDF). According to Table 5 (see PDF). The mean Distance between the RMF and the origin of the nerve for males is 7.7112 ± 8.23832 mm and for females is 6.7561 ± 8.30169 mm. The mean Distance between the RMF and the Third molar for males is3.1138 ± 3.75423mm and for females is 2.5677± 3.36780 mm.

## Discussion:

This study evaluated the incidence and anatomical properties of the RMC using CBCT images and presented a comprehensive classification of the RC types by reviewing the previous studies. In clinical practice, anatomical variations, such as supplemental or accessory canals and foramina, can only be detected by radiologic methods. However, conventional two-dimensional (2D) radiographs such as panoramic images are insufficient for detecting all anatomical structures and in particular the presence of an RMC [16]. The current study was aimed to identify the incidence of retromolar canal in the mandible using CBCT, scans with 0.5 mm voxel size. Several methods have been employed in the past to evaluate the RMC. Previous studies have reported considerable variation in the prevalence of the RMC (0-72%) [7, 17]. The results were influenced by the population studied, sample size and methodology used for evaluation [cadavers, dried mandibles, panoramic radiography, CBCT etc. A majority of these earlier studies have evaluated the prevalence of RMC on dried mandibles and on cadavers, but relatively few studies have been conducted in living subjects [using panoramic radiographs, computed tomography CT and CBCT. That significant discrepancy may be due to the lack of a 3D representation of the canal system. Since these investigations only looked at the bone surface without sectioning, they were not linked to any RMC categorization. As a result, it is possible that some of these foramina were unrelated to the RMCs. Due to panoramic studies' limitations as a 2D modality; it is evident that the incidence rates reported in CBCT studies and panoramic studies varied significantly; many investigations have recovered incidence rates of 7.3-75.4% [18] and 3-8% [19]. In our study, the RMC was present in 64 of 101 CBCT images (63.3%). The high incidence of the RMC in this study compared with previous studies may be explained by restrictions associated with the inclusion criteria applied in this present study (only with the third molars) and other radiological variables such as a bigger voxel size (0.5mm; (compared with voxel sizes of 0.08-0.125 mm in previous studies). Numerous CBCT studies have been conducted on the mandible the incidence rates range from 25.4 to 75.4% and 8.5 to 17.4%, respectively. According to Freitas GB et al 2017 of the 300 CFCT scans analyzed, a single mandibular canal was observed in 210 (70.0%). In the remaining 90 cases, anatomical changes were observed relating to this canal, indicating that the prevalence of this condition in this sample was 30%. The prevalence of retromolar canals was observed in 15 patients (5.0%), [20] which is not in accordance to our study.

Von Arx *et al.* 2011 [21] provided a detailed description of the courses, morphologies and dimensions of 31 RMCs on 121 mandibular sides (25.6%). The mean Distance between the RMF and the Third molar for males is 3.1138 ± 3.75423mm and for females is 2.5677 ± 3.36780 mm, which was comparable to the results of Han *et al.* who found the distance to be 14.08 ± 3.85, von Arx *et al.* who found the distance to be 15.16 ± 2.39 mm [21] Accordind to literature review by Filo *et al.* 2015 Seven of the 33 cited studies present percentages of RMF in the right and left sides of the mandible. Four studies demonstrate right-sided prevalence, two studies demonstrate left-sided prevalence and one study with two separate populations demonstrates equal-sided prevalence and left-sided prevalence. Three studies cite no side predilection [22]. Kawai *et al.* 2016 [23] described the frequency, location and content of 47 RMFs on 140 mandibular sides (37%) A variety of factors, including racial, genetic and environmental ones, can account for this large variation in incidence rates. The sample size may possibly be responsible for the large variation in the incidence rate of RMC observed in various studies. It was discovered that there were more unilateral canals than bilateral canals, the bulk of which were located on the mandibular left side, which was same as this study. These measurements may aid the clinician to better localize the RMC in various clinical procedures. Thus, the 3rd molars can be used as anatomical landmarks, hence saving the surgeon from the complication of damaging the retromolar nerve and its associated morbidity. Regarding the measurements, the current study found the mean Distance between the RMF and the origin of the nerve for males is 7.7112 ± 8.23832 mm and for females is 6.7561 ± 8.30169 mm. The method for classifying RMCs proposed by Patil *et al.* [24] has been used in this investigation. It proved to be the most practical classification. According to the course of the canals, the most common type in our study was type C 56.63%, followed by type A 21% and type B was the rarest type with only 8%. These results are different from Patil *et al.*'s study that found type B to be the most common. This can be explained by the voxel size used in their study (0.08 mm) which allowed them to investigate much narrower canals and most of the type B canals in Patil *et al.*'s study are of very small diameter [24]. Park *et al.* and Ogawa *et al.* found the distance between the RMF and the third molar to be 5.8 ± 3.6mm and 5.5 ± 2.1, respectively, which are not much different from our findings of 4.26 ± 4.21mm [25]. The system can also be used to assess the characteristics of the RMC/RMF both qualitatively and quantitatively in future.

## Inference:

The study demonstrated that the retromolar canal (RMC) is a common anatomical variation in the mandible, with an incidence rate of 63.3% per patient. Type C RMC was the most prevalent pattern observed. The findings emphasize the variability of RMC morphology and its frequent occurrence on both sides, highlighting the clinical significance of preoperative CBCT evaluation.

## Conclusion:

CBCT effectively identifies the retromolar canal and its variants, helping clinicians prevent surgical complications during mandibular procedures.Preoperative CBCT assessment is essential to avoid nerve injury, bleeding, and anesthetic failure in the retromolar region.
